# Evaluation of an AI-based, automatic coronary artery calcium scoring software

**DOI:** 10.1007/s00330-019-06489-x

**Published:** 2019-11-14

**Authors:** Mårten Sandstedt, Lilian Henriksson, Magnus Janzon, Gusten Nyberg, Jan Engvall, Jakob De Geer, Joakim Alfredsson, Anders Persson

**Affiliations:** 1 Center for Medical Image Science and Visualization (CMIV), Linköping University, Linköping, Sweden; 2 Department of Radiology and Department of Medical and Health Sciences, University Hospital of Linköping, Linköping University, SE-581 85 Linköping, Sweden; 3 Department of Cardiology and Department of Medical and Health Sciences, Linköping University, Linköping, Sweden; 4 Department of Clinical Physiology and Department of Medical and Health Sciences, Linköping University, Linköping, Sweden

**Keywords:** Artificial intelligence, Software, Coronary artery disease, Multidetector computed tomography

## Abstract

**Objectives:**

To evaluate an artificial intelligence (AI)–based, automatic coronary artery calcium (CAC) scoring software, using a semi-automatic software as a reference.

**Methods:**

This observational study included 315 consecutive, non-contrast-enhanced calcium scoring computed tomography (CSCT) scans. A semi-automatic and an automatic software obtained the Agatston score (AS), the volume score (VS), the mass score (MS), and the number of calcified coronary lesions. Semi-automatic and automatic analysis time were registered, including a manual double-check of the automatic results. Statistical analyses were Spearman’s rank correlation coefficient (⍴), intra-class correlation (ICC), Bland Altman plots, weighted kappa analysis (κ), and Wilcoxon signed-rank test.

**Results:**

The correlation and agreement for the AS, VS, and MS were *⍴* = 0.935, 0.932, 0.934 (*p* < 0.001), and ICC = 0.996, 0.996, 0.991, respectively (*p* < 0.001). The correlation and agreement for the number of calcified lesions were *⍴* = 0.903 and ICC = 0.977 (*p* < 0.001), respectively. The Bland Altman mean difference and 1.96 SD upper and lower limits of agreements for the AS, VS, and MS were − 8.2 (− 115.1 to 98.2), − 7.4 (− 93.9 to 79.1), and − 3.8 (− 33.6 to 25.9), respectively. Agreement in risk category assignment was 89.5% and κ = 0.919 (*p* < 0.001). The median time for the semi-automatic and automatic method was 59 s (IQR 35–100) and 36 s (IQR 29–49), respectively (*p* < 0.001).

**Conclusions:**

There was an excellent correlation and agreement between the automatic software and the semi-automatic software for three CAC scores and the number of calcified lesions. Risk category classification was accurate but showing an overestimation bias tendency. Also, the automatic method was less time-demanding.

**Key Points:**

*• Coronary artery calcium (CAC) scoring is an excellent candidate for artificial intelligence (AI) development in a clinical setting.*

*• An AI-based, automatic software obtained CAC scores with excellent correlation and agreement compared with a conventional method but was less time-consuming.*

## Introduction

Non-contrast-enhanced, ECG-triggered, coronary calcium scoring computed tomography (CSCT) detects coronary artery calcifications (CAC) at low radiation doses [1] and is reliable in predicting future cardiovascular (CV) events for asymptomatic patients, independent of conventional risk models [2]. Clinical guidelines in the USA [3, 4] and Europe [5] recommend CSCT in selected asymptomatic individuals, typically with an intermediate probability in a pre-test, clinical CV risk assessment.

The most commonly used CSCT technique for CAD grading and risk is the Agatston score (AS), other techniques are the volume score (VS) and the mass score (MS) [6].

The CAC scoring is traditionally performed by experts using semi-automatic software’s which includes manual identification and marking of the calcified coronary artery lesions. Guidelines worldwide endorse the use of CSCT, and the use of the method is likely to increase. Consequently, there is a need for more efficient automatic systems. The last few years have brought improvements in artificial intelligence (AI) radiology systems. A recent study in CT diagnostics of lung cancer, for example, demonstrated AI to be on par with or even outperforming radiologists [7]. In CAC scoring, AI could have a similar potential to assist or replace the human reader, thereby reducing clinical workload and increasing efficiency.

The aim of the study presented herein was to compare an automatic AI-based CSCT post-processing software prototype to a semi-automatic, conventional software by evaluating the correlation and agreement of the total AS, VS, MS, and the number of calcified coronary lesions. Also, comparison for CV-based risk classification into five commonly used categories was performed. Finally, time for analysis was evaluated.

## Material and methods

### Ethics

This study was conducted according to the principles set forward in the declaration of Helsinki and according to Good Clinical Practice. Permission was obtained from the regional ethical review board (Dnr 2018/535-32). In accordance with the ethical regulations for Swedish registries and Swedish legislation, patients were informed about their participation in a registry, and the right to deny participation or have data removed, which waivers any requirements for written consent.

For the multi-hospital acquired training data, necessary ethics approval and/or patient consent was obtained when required.

### Study sample

**Fig. 1:**
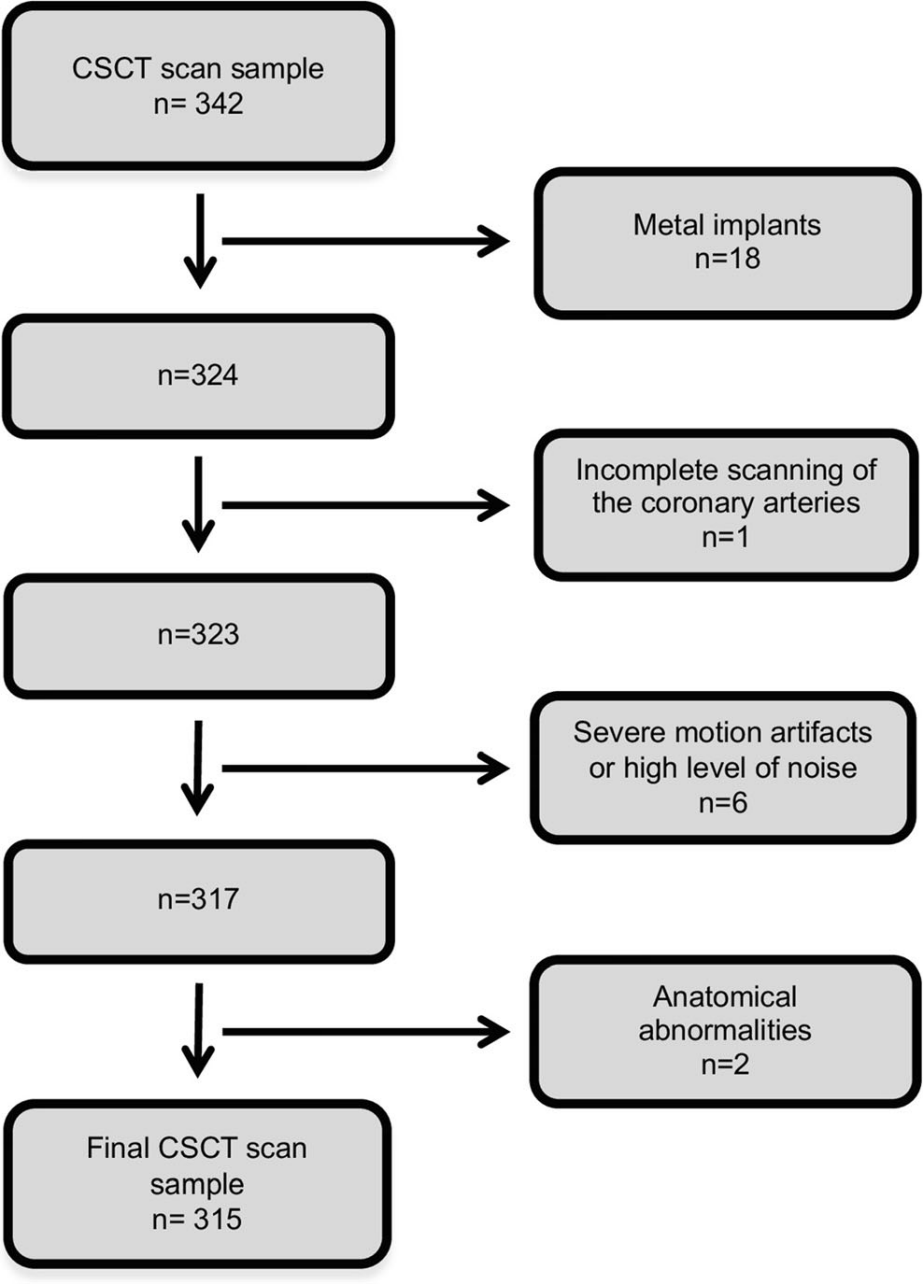
Flow chart. Inclusions and exclusions

In this observational, cross-sectional study, patients and their baseline characteristics were retrospectively collected from a nationwide quality registry, SWEDEHEART [8]. All patients with a CSCT performed on a particular state-of-the-art CT scanner between 1 December 2017 and 31 January 2019 (*n* = 342) at Linköping University Hospital were consecutively included. The indication was suspected ischemic heart disease. Exclusion criteria were, as previously suggested [9], anatomical abnormalities (*n* = 2), intracoronary stents (*n* = 0), metal implants (*n* = 18), and CSCT scans with severe motion artifacts or high level of noise determined by visual inspection (*n* = 6) (Fig. 1). In addition, one CSCT scan (*n* = 1) was excluded due to incomplete scanning of the coronary arteries. The final main dataset consisted of 315 CSCT scans.

In total, 13 out of 20 CSCT with anatomical abnormalities and metal implants were considered readable for CAC scoring, and represented an independent dataset (*n* = 13).

### CT acquisition parameters and image reconstruction

All CSCT scans were acquired through the use of a Siemens SOMATOM Force (Siemens Healthineers) MDCT. A prospectively ECG-triggered high-pitch spiral CSCT scan was performed, with a tube voltage of 120 kV, and automated tube current modulation (CARE Dose4D, Siemens) with a setting of 40 quality ref. mAs. Further settings were as follows: gantry rotation time 0.25 s, pitch 3.2, collimation 192 × 0.6 mm, matrix size 512 pixels, and temporal resolution 66 ms. The scan was set to start at 65% of the cardiac cycle. Reconstructions were made with a routine weighted filtered back projection (WFBP, Siemens) algorithm using medium sharp convolution kernel (Qr36), 3.0 mm section thickness, and increment 1.5 mm. Beta blockers were administered if the heart rate was > 65 bpm. After CTCS scanning, a CCTA was performed in the same session.

### AI-based, automatic system overview

The automatic software was trained on multi-vendor, multi-scanner, and multi-hospital, anonymized data from routine coronary calcium scoring acquisitions. No training datasets were used in the current study.

During model training, the locations of the coronaries created a territory map in a heart-centric coordinate system. This map serves to assign prior likelihood of different voxels belonging to the coronary arteries.

For each evaluated CSCT scan, a model is used to segment the heart, to establish a heart-centric coordinate system. The pre-computed coronary territory weights are mapped to the local size and shape of the patient’s heart. All voxels > 130 HU are extracted. Around each voxel, an image patch is extracted to represent the local spatial characteristics, the prior likelihood from the territory map and the location (x, y, z) of the voxel in the heart-centric coordinate system. The model uses these features to make a prediction that this voxel belongs to the coronaries.

Some work [10–12] already used patient-specific, heart-centric coordinate systems, but relied on manually placed markers, or local image coordinates in combination with a computationally expensive registration to an atlas-based model [13, 14]. Another work used a heart segmentation but no further classification besides voxel intensity [15]. As far as we know, the evaluated new machine learning model that combines the location within this coordinate system, the local image information around a voxel, and the coronary territory map is novel.

### Data reporting

A standard reference was obtained with a semi-automatic, previously validated [16], post-processing software (syngo.via, Siemens Healthineers). All 315 CSCT scans were double read by two radiologists in at least two sessions (M.S. and S.S., both with 10 years’ experience of cardiac CT reading) and all interpretation differences were resolved by consensus. To determine the presence of CAC, an attenuation threshold was set at > 130 HU. Calcified coronary objects having an area of ⋝ 1 mm^2^ were included, as originally described [17] using default software settings. Every calcified region of interest was manually identified and marked to attain the total AS, VS, MS, and the number of calcified coronary lesions. The time used for the first read was registered.

A total of 62 (20%) CSCT scans from the standard reference underwent a second opinion evaluation from two additional readers (A.P., radiologist, 20 years’ experience of cardiac CT reading, and L.H., cardiac imaging radiographer, 2 years’ experience of cardiac CT research reading). CSCT scans selected for second opinion were those considered to potentially shift in risk category due to readers arbitrariness (*n* = 32), calcifications close to the coronary ostia (*n* = 27), or difficulties to discriminate peripheral calcified coronary lesions from noise (*n* = 3). After consensus was reached, two changes were made, both with AS difference ≤ 5, and there was no shift in risk category.

Based on the AS, CSCT scans were assigned into commonly used risk groups with cutoff values: CAC 0: No identifiable plaque, very low CV. CAC 1–10: Minimal plaque burden, low CV risk. CAC 11–100: Mild atherosclerotic plaque burden, moderate CV. CAC 101-400: At least moderate atherosclerotic plaque burden, moderately high CV risk. CAC > 400: Extensive atherosclerotic plaque burden, high CV risk [18].

For inter-reader agreement, a subset of 106 (33.6%) CSCT scans were randomly selected and assigned to two independent radiologists. One radiologist was assigned 71 CSCT scans (G.N., 1 year experience of cardiac CT reading) and one radiologist 35 CSCT scans (A.B., 16 years’ experience of cardiac CT reading), both blinded to previous results.

The automatic software was implemented in MeVisLab on a regular workstation. All CSCT scans (*n* = 315) were analyzed with the automatic software, retrieving the total AS, VS, MS, and number of calcified coronary lesions. The automatic system run-time and the time for a manual double-check of the results were registered. The double-check included a localization of all CAC, and to attain an image-based numerical correlation to the automatically derived number of calcified coronary lesion.

**Fig. 2:**
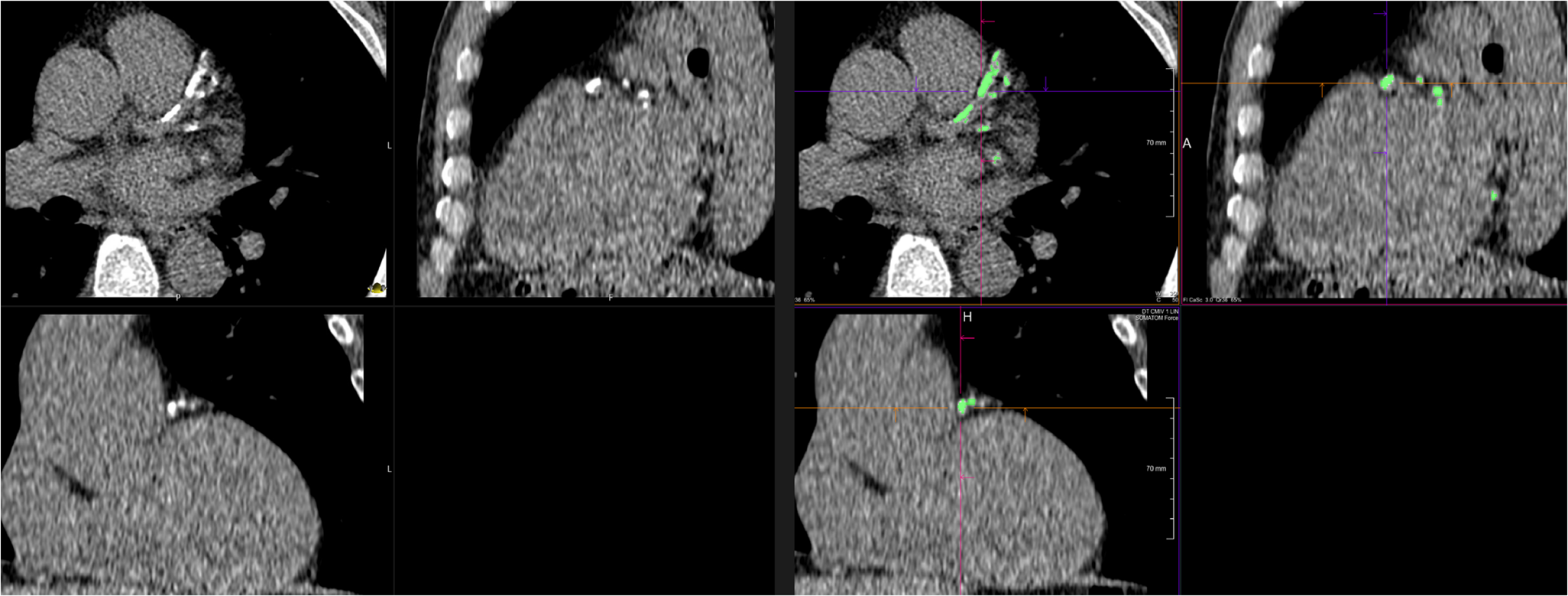
Multiplanar reconstructions of a coronary calcium scoring computed tomography scan with extensive calcifications in left anterior descending artery and circumflex artery. Left: Pre-processing images demonstrating calcified lesions as high attenuation regions. Right: Post-processing, visual coronary calcium scoring feedback. High attenuation regions that contributed to the results are displayed in green color

No human interaction was needed, except for loading data into the software. For each CSCT scan, a visual CSCT feedback with crosshairs was displayed in three dimensions, allowing multiplanar reconstructions (Fig. 2).

The readable CSCT scans (*n* = 13) with coronary abnormalities and metal implants were independently reported, following the same routine. However, another radiologist (G.W., 16 years’ experience of cardiac CT reading) performed the semi-automatic double-read, and there was no second opinion.

### Statistics

Continuous data are presented as mean ± standard deviation if normally distributed, or as median and interquartile range (IQR) if non-normally distributed. Categorical data are presented as numbers and percentages. Normality was tested with Shapiro-Wilk’s test. The correlation and agreement between the standard reference and the automatic software for the AS, VS, MS, and the number of lesions were assessed with Spearman’s rank correlation coefficient (*⍴*) and intraclass correlation coefficient (ICC), as appropriate for non-parametric data. Bland Altman plots displayed bias and limits of agreements within 95% confidence interval. Differences in risk classifications were assessed by weighed kappa analysis (κ) and accuracy. Inter-observer agreement was demonstrated with ICC and Spearman’s rank correlation coefficient (*⍴*). Difference in time was analyzed with Wilcoxon signed-rank test. A two-sided *p* < 0.05 was considered statistically significant. Randomization for inter-rater agreement was achieved by Excel (Microsoft Office 365); all other analyses were performed using IBM SPSS v.24 (IBM SPSS).

## Results

In total, 315 patients were included based on inclusion/exclusion criteria, 171 (54.3 %) women and 144 (45.7%) men. Mean age was 58 years (± 11.5 SD). All baseline characteristics are presented in Table 1.

**Table 1:** Baseline characteristics

All patients (*n*)	315
Women (*n* (%))	171 (54.3)
Men (*n* (%))	144 (45.7)
Age (years)	58 (± 11.5)
Body mass index (kg/m^2^)	27.3 (± 4.7)
Hypertension (*n* (%))	164 (52.1)
Diabetes (*n* (%))	26 (8.3)
Hyperlipidemia (*n* (%))	86 (27.3)
Non-smokers (*n* (%))	165 (52.4)
Smokers (*n* (%))	32 (10.2)
Ex-smokers, > 1 month (*n* (%))	117 (37.1)

Continuous variables are presented as mean ± standard deviation

**Fig. 3:**
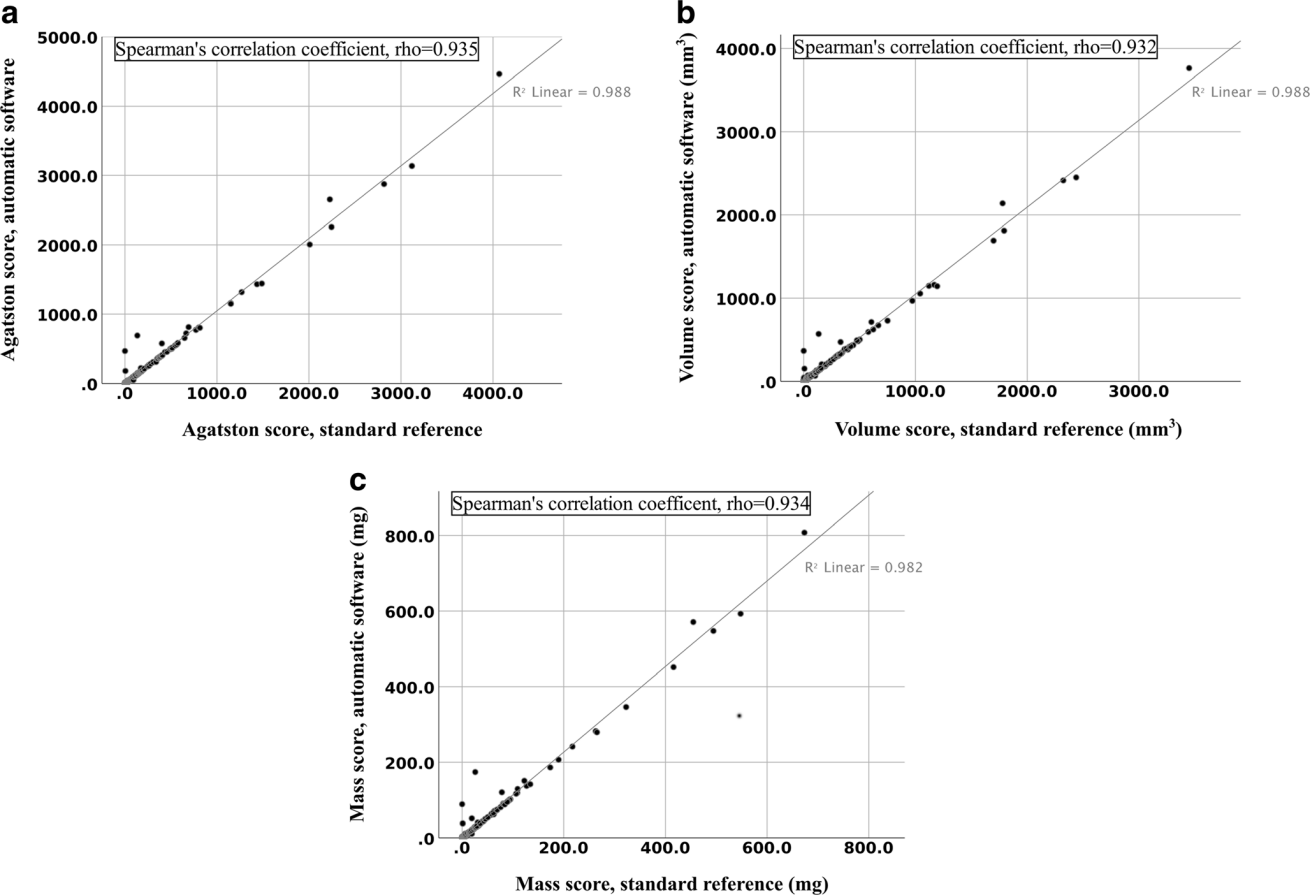
Scatter plot depicting coronary calcium score correlation between the standard reference and automatic software, expressed as Spearman’s rank correlation coefficient, rho (*⍴*). **a** Agatston score; **b** Volume score; **c** Mass score

The correlation for the automatic software in relation to the standard reference with respect to the AS, VS, and MS was assessed with the Spearman’s rank correlation coefficient showing *⍴* = 0.935, 0.932, and 0.934 (*p* < 0.001), respectively (Fig. 3).

The agreement for the automatic software in relation to the standard reference with respect to the AS, VS, and MS was assessed with the ICC, showing 0.996, 0.996, and 0.991, respectively (*p* < 0.001).

**Fig. 4:**
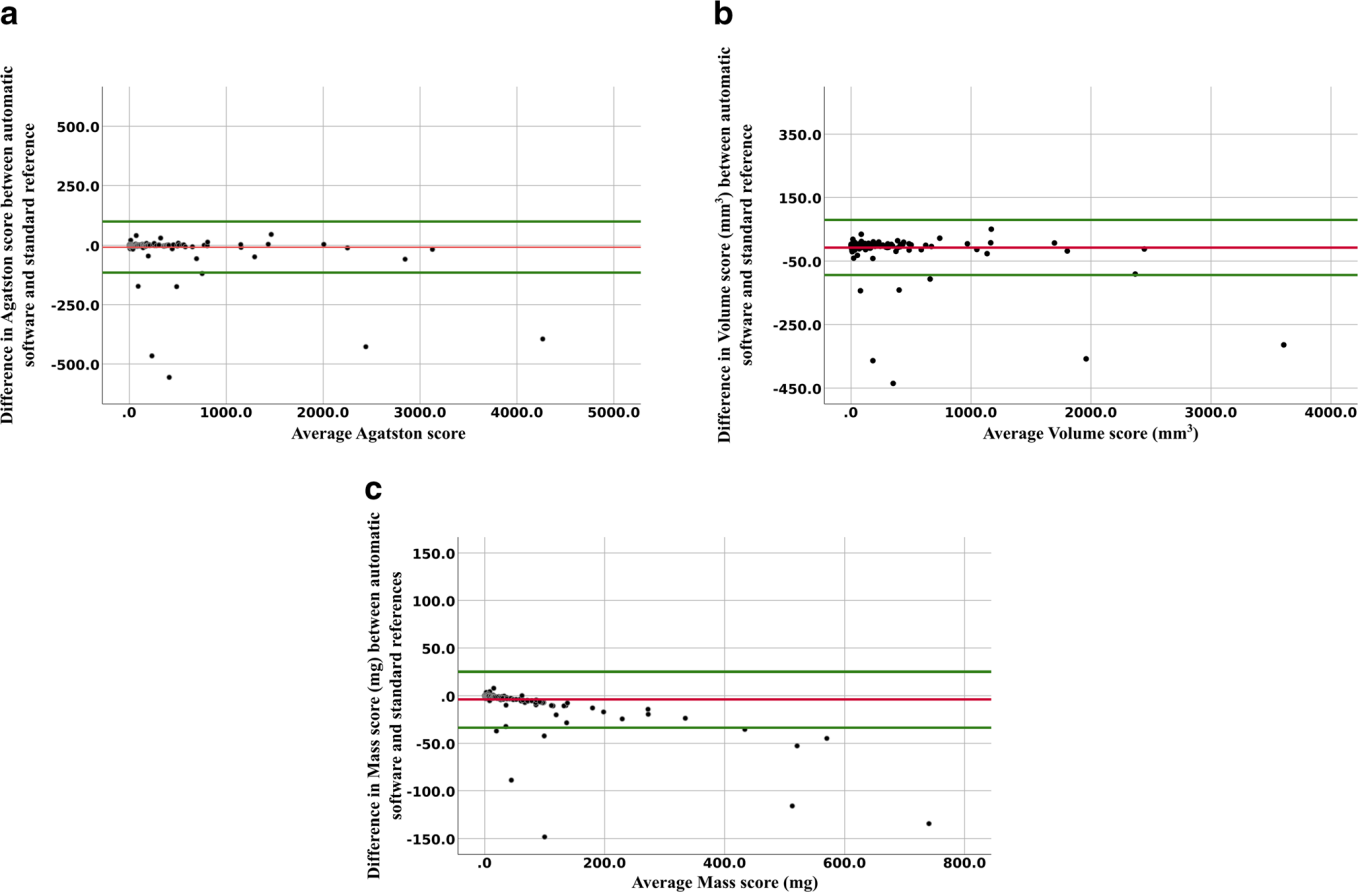
Bland Altman analyses depicting difference in coronary calcium score between automatic software and standard reference, plotted against mean of the coronary calcium score measurements. Mean difference is in red; upper and lower limits of agreement with 95% confidence interval are in green. **a** Agatston score: mean difference − 8.2 and limits of agreement − 115 to 98.2. **b** Volume score: mean difference − 7.2 and limits of agreement − 93.9 to 79.1. **c** Mass score: mean difference − 3.8 and limits of agreement − 33.6 to 25.9

Bland Altman plots mean difference and 1.96 SD upper and lower limits of agreements were as follows: AS − 8.2 (− 115.1 to 98.2), VS − 7.4 (− 93.9 to 79.1), and MS − 3.8 (− 33.6 to 25.9) (Fig. 4). A few outliers contributed to an overestimation tendency of CAC scores by the automatic software, mostly in the lower ranges.

A confusion matrix for risk category assignment demonstrated an accuracy of 89.5% and weighed kappa analysis (κ) = 0.919 (*p* < 0.001) (Table 2). In total, 33 CSCT scans were misclassified: 27 were overestimated and six underestimated. In total, 29 CSCT scans were off by one category, 19 of those shifting from AS 0 to AS 1–10. All the 19 CSCT scans shifting from AS 0 to AS 1–10 were due to erroneous registration of image noise in the heart and/or adjacent structures, 13 had an AS difference < 2, and four had an AS difference between two and eight. Three CSCT scans were off by two categories, two due to inclusions of aortic root calcifications, and one due to inclusion of a pericardial calcification. One CSCT scan was off by four categories due to inclusion of a mitral valve calcification. All underestimated CSCT scans were off by one category, four had an AS difference of ≤ 2 and one had an AS difference of 21, the latter not including a calcification close to the right coronary ostium.

**Table 2:** Confusion matrix

Standard reference	Automatic software									
	Category	0	1–10	11–100	101–400	> 400	Total	Same	Shift up	Shift down
	0	*119*	19	2	0	1	141	*119*	22	-
	1–10	4	*34*	3	1	0	42	*34*	4	4
	11–100	0	1	*55*	0	0	56	*55*	0	1
	101–400	0	0	0	*43*	1	44	*43*	1	0
	> 400	0	0	0	1	*31*	32	*31*	0	1
	Total	123	54	60	45	33	*315*			

Confusion matrix with distribution of cardiovascular disease (CVD) risk categorization comparing the standard reference with the automatic software. Accuracy = 89.5% and weighed kappa analysis (κ) = 0.919 (*p* < 0.001). Columns to the right demonstrate a summary of risk category shifting. No risk category shifting is indicated in italics

The correlation and agreement for the automatic software in relation to the standard reference with respect to the number of calcified coronary lesions were assessed with the Spearman’s rank correlation coefficient and the ICC showing *⍴* = 0.903 and ICC = 0.977 (both with *p* < 0.001), respectively.

The correlation and agreement for inter-reader agreement between the standard reference and the independent readers with respect to AS were assessed with the Spearman’s rank correlation coefficient and the ICC, showing *⍴* = 0.968 and ICC = 1.000 (both with *p* < 0.001), respectively.

The correlation and agreement for the automatic software in relation to the independent readers with respect to AS were assessed with the Spearman’s rank correlation coefficient and the ICC, showing *⍴* = 0.909 and ICC = 0.979 (both with *p* < 0.001), respectively.

The median time to obtain the semi-automatic reading was 59 s (IQR 35–100), compared with automatic run-time 9 s (IQR 5–18), and 36 s (IQR 29–49) (*p* < 0.001) for the automatic run-time plus double-checking the results.

Among the separate 13 CSCT scans having coronary abnormalities and metal implants, the correlation and agreement between the standard reference and the automatic software with respect to AS were assessed with the Spearman’s rank correlation coefficient and the ICC, showing *⍴* = 0.939 and ICC = 0.956 (both with *p* < 0.001), respectively. Risk category assignment demonstrated an accuracy of 54% and weighed kappa analysis (κ) = 0.621 (*p* = 0.001).

## Discussion

In this study, assessment of three CAC scores and number of calcified coronary lesions obtained from an AI-based, automatic post-processing software were evaluated using a semi-automatic post-processing software as a reference. Correlation, agreement, and subsequent risk classification were excellent, and the automatic analysis was less time-consuming.

The correlation and agreement of the AS, VS, and MS of the automatic software compared with the standard reference was excellent. The Bland Altman plot for the AS, VS, and MS demonstrated narrow limits of agreement, but a small overestimation bias in the lower range of CAC scores. The risk group categorization was accurate, yet 33 (10.5%) CSCT scans were misclassified, the majority shifting from AS 0 to AS 1–10. This misclassification bias in the lowest AS score could be a clinical shortcoming, since AS > 0 is suggested to represent incipient CAD, and may therefore be a gatekeeper for prescription of medication [19–21]. Notably, this bias in the low AS score range may be exaggerated due to skewness in the dataset, since 140 out of 315 CSCT scans (44%) had an AS 0*.* All CSCT scans off by two or more categories were due to errors not likely to be made by experts, possibly reflecting AI-specific challenges. However, these false CAC results are recognizable in the visual CSCT feedback, therefore unlikely to be clinically problematic.

There was an excellent correlation and agreement regarding the number of calcified coronary lesions, a feature previously demonstrated to have a prognostic value [22].

The number of studies evaluating automatic CSCT software is limited, and comparisons with other studies are difficult due to differences in study designs, inclusions, image acquisition, reconstructions, and quantitative evaluation methods. However, the correlation and agreement for CAC scoring and risk category classification were overall in line with previous studies having roughly similar prerequisites [12–14]. Automatic systems applied on the orCaScore framework have demonstrated excellent result [9, 23], but the orCaScore dataset has an even distribution amongst risk categories and is smaller. Also, CAC scoring is mainly applied to asymptomatic patients, probably more likely to have a CAC distribution similar to this study.

Substantial efforts were made in creating a strong standard reference, both with double readings and a subset of CSCT scans for second opinion. A close to perfect inter-reader agreement was achieved, which is not unique for this study [13, 14, 24], but it indicates an adequate reliability. One radiologist assigned CSCT scans for inter-reader agreement was relatively inexperienced, but the results were still excellent, possibly indicating that CAC scoring is not particularly difficult. The fact that CSCT requires expert interaction, yet is relatively simple, makes it an excellent candidate for AI development in a clinical setting. However, an acceptable run-time is an important prerequisite, being congruent with, or slightly faster than what has previously been described [23]. While the automatic software is faster, it also takes expert time to confirm the results. Therefore, the automatic run-time plus the reader confirmation time may be a clinically more appropriate registration, in this study still shorter than the semi-automatic method. However, the CAC scoring feedback is not editable. When necessary, the time for corrections could therefore not be registered. Considering this study’s risk group classification accuracy, > 1/10 of the examinations would have needed a manual correction, inevitably to the cost of additional time.

Interestingly, 48% of the excluded CSCT scans were still applicable for semi-automatic reading, showing an excellent correlation and agreement compared with the automatic software but demonstrating less accurate risk group categorization. However, this should be analyzed in larger samples.

No data were missing, but there are limitations to this study. First, all CSCT scans were conducted at a single center on only one CT scanner. This could be a shortcoming since the AS derived from ex vivo human hearts examined on different CT vendors have variations [25]. Yet, there were no substantial differences in inter-scan variability in an in vivo study with 30 patients [24]. Second, the automatic software is only compared with a semi-automatic software from the same vendor. However, this semi-automatic software was previously compared with other vendors, demonstrating similar results [16], thereby supporting external validity. Third, a total of 27 CSCT scans (8.6 %) were excluded, limiting generalizability. Fourth, this study is limited to numerical correlation and agreements; no comparisons were performed for individual CSCT scans. Possible false positive and false negative high attenuation objects are thus not evaluated. Fifth, all CSCT scans were obtained from symptomatic patients with no known ischemic heart disease, while the technique is more commonly applied on asymptomatic patients. However, the vast majority had low pre-test probability and a large proportion of no detectable CAD, therefore probably similar to an asymptomatic population. Sixth, time for semi-automatic reading did not include the double read or the second opinion. Nonetheless, CAC scoring is usually performed by one single reader. Seventh, time used for CSCT reading may have inter-reader variations, and our registrations were derived from only one radiologist. Eighth, in the automatic time registration double-check, numerical correlations were not applicable if extensive CAC, due to indistinguishable calcified lesions. Lastly, a larger dataset could reach statistically stronger results. Still, with one exception [14], this is the largest known study evaluating automatic CAC scoring derived from CSCT scans.

## Conclusion

There was an excellent correlation and agreement between the automatic and the reference standard for three CAC scores and for the number of calcified coronary lesions. Risk category classification was accurate, but with an overestimation bias tendency, especially when AS was zero. Double-checking the results should therefore be mandatory. Nonetheless, the automatic run-time plus a manual double-check of the results were still less time-consuming than using the reference standard.

## Abbreviations

AI: Artificial intelligence
AS: Agatston score
CAC: Coronary artery calcification
CAD: Coronary artery disease
CCTA: Computed coronary tomography angiography
CSCT: Calcium scoring computed tomography
CT: Computed tomography
CV: Cardiovascular risk
CVD: Cardiovascular disease
EBCT: Electron beam computed tomography
ICC: Intraclass correlation coefficient
IQR: Interquartile range
MDCT: Multidetector computed tomography
MS: Mass score
SD: Standard deviation
VS: Volume score

## References

[1] Apfaltrer G, Albrecht MH, Schoepf UJ (2018). High-pitch low-voltage CT coronary artery calcium scoring with tin filtration: accuracy and radiation dose reduction. Eur Radiol.

[2] Detrano R, Guerci AD, Carr JJ (2008). Coronary calcium as a predictor of coronary events in four racial or ethnic groups. N Engl J Med.

[3] Goff DC, Lloyd-Jones DM, Bennett G (2014). 2013 ACC/AHA guideline on the assessment of cardiovascular risk: a report of the American College of Cardiology/American Heart Association Task Force on Practice Guidelines. Circulation.

[4] Greenland P, Alpert JS, Beller GA (2010). 2010 ACCF/AHA guideline for assessment of cardiovascular risk in asymptomatic adults: executive summary: a report of the American College of Cardiology Foundation/American Heart Association Task Force on Practice Guidelines. Circulation.

[5] Piepoli MF, Hoes AW, Agewall S et al (2016) 2016 European Guidelines on cardiovascular disease prevention in clinical practice: The Sixth Joint Task Force of the European Society of Cardiology and Other Societies on Cardiovascular Disease Prevention in Clinical Practice (constituted by representatives of 10 societies and by invited experts): Developed with the special contribution of the European Association for Cardiovascular Prevention & Rehabilitation (EACPR). Eur J Prev Cardiol 23:NP1–NP96 10.1177/2047487316653709 27353126

[6] Alluri K, Joshi PH, Henry TS, Blumenthal RS, Nasir K, Blaha MJ (2015). Scoring of coronary artery calcium scans: history, assumptions, current limitations, and future directions. Atherosclerosis.

[7] Ardila Diego, Kiraly Atilla P., Bharadwaj Sujeeth, Choi Bokyung, Reicher Joshua J., Peng Lily, Tse Daniel, Etemadi Mozziyar, Ye Wenxing, Corrado Greg, Naidich David P., Shetty Shravya (2019). End-to-end lung cancer screening with three-dimensional deep learning on low-dose chest computed tomography. Nature Medicine.

[8] Jernberg T, Attebring MF, Hambraeus K (2010). The Swedish Web-system for enhancement and development of evidence-based care in heart disease evaluated according to recommended therapies (SWEDEHEART). Heart.

[9] Wolterink JM, Leiner T, de Vos BD (2016). An evaluation of automatic coronary artery calcium scoring methods with cardiac CT using the orCaScore framework. Med Phys.

[10] Kurkure U, Chittajallu DR, Brunner G, Le YH, Kakadiaris IA (2010). A supervised classification-based method for coronary calcium detection in non-contrast CT. Int J Cardiovasc Imaging.

[11] Brunner G, Chittajallu DR, Kurkure U, Kakadiaris IA (2010). Toward the automatic detection of coronary artery calcification in non-contrast computed tomography data. Int J Cardiovasc Imaging.

[12] Shahzad R, van Walsum T, Schaap M (2013). Vessel specific coronary artery calcium scoring: an automatic system. Acad Radiol.

[13] Wolterink JM, Leiner T, Takx RAP, Viergever MA, Išgum I (2014) An automatic machine learning system for coronary calcium scoring in clinical non-contrast enhanced ECG-triggered cardiac CT. SPIE vol 9035, pp 90350 10.1109/TMI.2015.2412651 25794387

[14] Wolterink JM, Leiner T, Takx RA, Viergever MA, Isgum I (2015). Automatic coronary calcium scoring in non-contrast-enhanced ECG-triggered cardiac CT with ambiguity detection. IEEE Trans Med Imaging.

[15] Isgum I, Rutten A, Prokop M, van Ginneken B (2007). Detection of coronary calcifications from computed tomography scans for automated risk assessment of coronary artery disease. Med Phys.

[16] Weininger M, Ritz KS, Schoepf UJ (2012). Interplatform reproducibility of CT coronary calcium scoring software. Radiology.

[17] Agatston AS, Janowitz WR, Hildner FJ, Zusmer NR, Viamonte M, Detrano R (1990). Quantification of coronary artery calcium using ultrafast computed tomography. J Am Coll Cardiol.

[18] Rumberger JA, Brundage BH, Rader DJ, Kondos G (1999). Electron beam computed tomographic coronary calcium scanning: a review and guidelines for use in asymptomatic persons. Mayo Clin Proc.

[19] Bittencourt MS, Blaha MJ, Blankstein R (2014). Polypill therapy, subclinical atherosclerosis, and cardiovascular events-implications for the use of preventive pharmacotherapy: MESA (Multi-Ethnic Study of Atherosclerosis). J Am Coll Cardiol.

[20] Pletcher MJ, Pignone M, Earnshaw S (2014). Using the coronary artery calcium score to guide statin therapy: a cost-effectiveness analysis. Circ Cardiovasc Qual Outcomes.

[21] Knudsen AD, Fuchs A, Kuhl JT (2018). Coronary artery calcium assessed with calibrated mass scoring in asymptomatic individuals: results from the Copenhagen General Population Study. Eur Radiol.

[22] Williams M, Shaw LJ, Raggi P (2008). Prognostic value of number and site of calcified coronary lesions compared with the total score. JACC Cardiovasc Imaging.

[23] Durlak Felix, Wels Michael, Schwemmer Chris, Sühling Michael, Steidl Stefan, Maier Andreas (2017). Growing a Random Forest with Fuzzy Spatial Features for Fully Automatic Artery-Specific Coronary Calcium Scoring. Machine Learning in Medical Imaging.

[24] Ghadri JR, Goetti R, Fiechter M (2011). Inter-scan variability of coronary artery calcium scoring assessed on 64-multidetector computed tomography vs. dual-source computed tomography: a head-to-head comparison. Eur Heart J.

[25] Willemink MJ, Vliegenthart R, Takx RA (2014). Coronary artery calcification scoring with state-of-the-art CT scanners from different vendors has substantial effect on risk classification. Radiology.

